# Additive transcriptomic variation associated with reproductive traits suggest local adaptation in a recently settled population of the Pacific oyster, *Crassostrea gigas*

**DOI:** 10.1186/s12864-015-1972-8

**Published:** 2015-10-19

**Authors:** Rossana Sussarellu, Arnaud Huvet, Sylvie Lapègue, Virgile Quillen, Christophe Lelong, Florence Cornette, Lasse Fast Jensen, Nicolas Bierne, Pierre Boudry

**Affiliations:** Ifremer, Laboratoire des Sciences de l’Environnement Marin UMR 6539 (UBO/CNRS/IRD/Ifremer), Plouzané, France; Ifremer, SG2M-LGPMM, Laboratoire de Génétique et Pathologie des Mollusques Marins, 17390 La Tremblade, France; UNICAEN, UMR BOREA MNHN, UPMC, UNICAEN, CNRS-7208, IRD207, F-14032 Caen, France; Fisheries and Maritime Museum, DK-6710 Esbjerg V, Denmark; Université Montpellier 2, Montpellier, France; CNRS - Institut des Sciences de l’Evolution, UMR5554, Station Méditerranéenne de l’Environnement Littoral, Sète, France; Present address: Ifremer, Laboratoire d’Ecotoxicologie, Nantes, France

**Keywords:** *Crassostrea gigas*, Adaptation, Invasiveness, Fertility, Sex-ratio, Transcriptome, Microarray

## Abstract

**Background:**

Originating from Northeast Asia, the Pacific oyster *Crassostrea gigas* has been introduced into a large number of countries for aquaculture purpose. Following introduction, the Pacific oyster has turned into an invasive species in an increasing number of coastal areas, notably recently in Northern Europe.

**Methods:**

To explore potential adaptation of reproductive traits in populations with different histories, we set up a common garden experiment based on the comparison of progenies from two populations of Pacific oyster sampled in France and Denmark and their hybrids. Sex ratio, condition index and microarray gene expression in gonads, were analyzed in each progeny (*n* = 60).

**Results:**

A female-biased sex-ratio and a higher condition index were observed in the Danish progeny, possibly reflecting an evolutionary reproductive strategy to increase the potential success of natural recruitment in recently settled population. Using multifarious statistical approaches and accounting for sex differences we identified several transcripts differentially expressed between the Danish and French progenies, for which additive genetic basis is suspected (showing intermediate expression levels in hybrids, and therefore additivity). Candidate transcripts included mRNA coding for sperm quality and insulin metabolism, known to be implicated in coordinated control and success of reproduction.

**Conclusions:**

Observed differences suggest that adaptation of invasive populations might have occurred during expansion acting on reproductive traits, and in particular on a female-biased sex-ratio, gamete quality and fertility.

**Electronic supplementary material:**

The online version of this article (doi:10.1186/s12864-015-1972-8) contains supplementary material, which is available to authorized users.

## Background

Invasive species are privileged models to analyse the evolution and adaptation of life history traits in new environments [1, 2] While an increasing number of studies have documented adaptation during invasion in terrestrial species [3, 4], few studies have been conducted in marine species to date [5–7]. Many marine species have a benthic sessile adult phase and disperse *via* a planktonic larval stage. In these organisms, high gene flow between populations can be ensured by high larval dispersal, high fecundity and huge population sizes [8, 9]. Nevertheless, paradoxical levels of genetic differentiation has been observed in marine populations [10, 11] originating from either local adaptation through natural selection or purely random processes such as genetic drift. In this context, marine invasive species offer the opportunity to study evolution and adaptation of life history traits upon introduction into new habitats*.*

Originating from Northeast Asia, the Pacific oyster *Crassostrea gigas* has been introduced and translocated worldwide, mainly for aquaculture purposes. Self-sustaining populations have today been recorded in at least 17 of them [12]. Although highly variable, the invasiveness of *C. gigas* has been demonstrated in several countries and this species is considered as pest or noxious in an increasing number of coastal areas [13]. In European waters, *C. gigas* is cultured from Norway to Portugal as well as in the Mediterranean Sea. This species rapidly settled along the Atlantic coasts of France following its massive introduction at the end of the 1960s [14]. More recently, feral populations of *C. gigas* have been reported in northern Europe [15, 16] as far north as Sweden, where dense populations of settled oysters can now be observed in several shallow water sites [17]. This expansion of Pacific oyster in the North Sea occurred much later after their first introduction than along the French Atlantic coast, suggesting that increase in population size may have been retarded by false or irregular recruitment depending on water temperature [15–18]. Irregular recruitment has been reported to coincide with above-average summer temperatures in late summer [19]. Indeed, *C. gigas* now reproduce and settle in Scandinavian waters as far as 60° N, while in the beginning of the 70’s, attempts of culture of this species failed at higher latitudes [18]. In this context, the success of the species and especially its northward expansion might be explained by climate change [20, 21] but also local adaptation as well as phenotypic plasticity, or both [22].

History of initial introductions and later transfers, natural connectivity resulting from natural dispersal and their resulting genetic structure are needed to assess the potential local adaptation of newly settled populations (for review see [6]). Moehler et al. [23] proposed that the genetic population structure of *C. gigas* in the Wadden sea could have been shaped by aquaculture practices. The presence of two separate genetic groups, one in the southern and one in the northern Wadden sea was suggested to be the result of two independent invasions. Rohfritsch et al. [24] investigated the possible effect of adaptation with a genome-scan approach. As previously reported [25, 26], no significant genetic structure was noted when comparing the population from Japan to European populations sampled along the French coasts and between populations of southern Europe. However, a significant genetic structure was observed in Europe among northern populations (located in Germany, Denmark and Sweden) and between northern and southern populations, together with lower genetic diversity in the north. Recently, Lallias et al. [27] highlighted how the number of oyster introduction events, aquaculture practices, genetic bottlenecks followed by genetic drift and natural dispersal could shape the genetic diversity and structure of introduced populations. Furthermore the analysis of F_ST_ outliers revealed 6 candidate loci for adaptation which could either reflect (i) parallel adaptation to similar environmental pressures (fjord-like environment) within each of the two groups or (ii) a footprint of a secondary introduction of an alternative genomic background, maintained by multifarious isolation factors [24]. This suggests that adaptation or reshuffling of pre-existing genetic backgrounds could have occurred during the invasion. In general, however, genome scans with an insufficient marker density have proved unsatisfactory to identify adaptation during marine invasion [24, 28], and the study of phenotypic differentiation has been proposed as a more promising approach [29, 30].

Colonization of new environments may promote rapid population divergence as a by-product of local adaptation to differential selective pressures [31]. Local adaptation in populations can be examined by analysing phenotypic traits that are likely to be differentially selected in the wild, but genetic bases of trait variation are necessary to allow response to natural selection. In most cases, the heritability estimates of these traits are low and their study requires the analysis under common conditions of progenies of wild genitors (rather than their direct study in the field), allowing disentangling environmental and genetic effects. In this context, gene expression studies can reveal adaptive mechanism, and the genetic basis of traits affecting fitness [32]. Furthermore, comparing the extent of quantitative genetic differences of phenotypic traits among populations could be assessed by the degree of differentiation in quantitative traits Q_ST_ [33] that has been widely used to assess the relative contributions of selection to phenotypic traits divergence [34, 35] and gene transcription profiles [36]. Transcriptomic scan (eQ_ST_) can indeed reveal adaptation, as outlier genes, showing the highest levels of differentiation between populations, will represent those that are most likely to evolve under directional selection [37]. Conversely, P_ST_ (differentiation phenotypic traits) and eP_ST_ scan can reveal selective selection [38]. In a context of climate change facilitating the establishment of invasive species, transcriptomic studies are promising in understanding basis of variation in phenotypic plasticity [39].

In this study we set out to investigate the evolutionary processes associated with the establishment and invasion into new environments of Pacific oyster population. Specifically, we raised progenies originating from Northern and Southern Europe along with their hybrids under common environmental conditions. A number of phenotypic traits, including gene expression patterns were analysed to search for evidence of differentiation in reproductive traits that might result from local adaptation.

## Methods

### Biological material

Wild oysters were collected in October 2009 at the Ile de Ré (France) and Limfjord (Denmark), transported to the Ifremer’s facilities in La Tremblade (Charente-Maritime, France) and kept in common controlled conditions. The sampling site in France is located 30 km from the location “MAR” that was studied by Rohfritsch et al. [24], while the sampling site in Limfjorden is identical to the location “LIM” [24]. Maturation was carried in shared conditions suitable for germ cell maturation according to Fabioux et al. [40] in Ifremer’s facilities in La Tremblade. In May 2010, crosses within and between populations were produced, using 40 parental individuals (20 females and 20 males) from each of the two populations. For the hybrid progeny, the same individuals were employed as parental oysters as for the within site ones, crossing French males with Danish females. Gametes were collected as described in Huvet et al. [41]. Briefly, sperm and oocytes were collected in seawater by stripping the gonads. For the two groups of females, oocytes were counted and equally pooled. Oocytes were then distributed in fertilization beakers and then fertilized separately by each male at a ratio of 100 spermatozoa/oocyte.

Progenies were reared under standard and common hatchery and nursery conditions. In October 2010, when about 10 mm large, juveniles were transferred to the field in the Marennes-Oléron basin until sampling in June 2011. For each progeny (“French”, “Danish” and “hybrid”), 100 oysters were randomly collected (in 4 replicated baskets) and weighed (total and wet flesh weights). The condition index, as specified by the French norm AFNOR was calculated using the following equation: (wet flesh weight/total weight) × 100 [42]. Gonads were immediately dissected from each oyster, a transversal section of the gonadic area was made for histological examination and the rest of the gonad was frozen in liquid nitrogen. Gonad tissues were crushed to a fine powder at −196 °C with an oscillating mill mixer and stored in liquid nitrogen until RNA extraction and biochemical analyses.

### Histological analysis

For each sample, a 3 mm cross-section of the visceral mass was excised in front of the pericardic region and immediately fixed in modified Davidson’s solution [43] at 4 °C for 48 h. Sex-ratio (i.e. the ratio males/males + females) and gonad developmental stage were determined by histological methods (see details in Fabioux et al. [40]) according to the reproductive scale of Steele and Mulcahy [44]. Sixty individuals for each progeny were chosen (for a total of 180) on the basis of their maturation stage (stage 3 of gametogenesis, ripeness) and sex for transcriptomic analysis.

### RNA extraction, amplification, labeling and microarray hybridization

Total RNA was isolated using 1.5 mL of Extract-all Reagent® (Eurobio AbCys, Courtaboeuf, France) *per* 50 mg of gonad powder, according to the manufacturer’s instructions. RNA concentrations were determined using a ND-1000 spectrophotometer (Thermo Scientific, Waltham MA, USA) at 260 nm, using the conversion factor 1 OD = 40 μg/mL RNA. RNA integrity was assessed on an Agilent bioanalyzer using RNA 6000 Nano kits® (Agilent Technologies, Santa Clara, CA, USA), according to manufacturer’s instructions. Over the 180 samples extracted, the RNA Integrity Number (RIN), obtained by setting the threshold “Unexpected Ribosomal Ratio” to 2 in the Agilent 2100 Bioanalyzer software considering the co-migration of 28S and 18S rRNA fragments in bivalves [45], varied from 7.9 to 9.6 (mean = 9.1 ± 0.3). For microarray hybridizations, 200 ng of total RNA were indirectly labelled with Cy3 using the Low Input Quick Amp Labeling kit One-Color® (Agilent Technologies), according to the manufacturer’s instructions. Qiagen RNeasy® (Quiagen, Venlo, Netherlands), mini spin columns were used for purifying amplified RNA samples. After purification, RNA amplification and dye incorporation rates were verified using a ND-1000 spectrophotometer (Thermo Scientific) and shown to lie between 100 and 200 ng/μL (RNA concentration) and between 1 and 5 pmol/μL RNA (dye incorporation). Hybridization was performed using the Agilent Gene expression hybridization kit® (5188–5242), as described by the manufacturer, with 1.65 μg of labelled RNA at 65 °C for 16 h. The employed arrays were Agilent 60-mer 4×44 K custom microarrays, containing 31,918 *C. gigas* transcripts, designed by Dheilly et al. [45]. Samples were randomly hybridized onto 48 different slides, which were subsequently treated with Gene expression wash buffer solution® (5188–5327; Agilent Technologies), Stabilization and Drying solutions® (5185–5979; Agilent Technologies). Slides were scanned on an Agilent Technologies G2565AA Microarray Scanner system® at 5 μm resolution, using default parameters. Features were extracted using the Agilent Feature Extraction software 6.1 (Agilent Technologies).

### Pre-processing and microarray data analysis

Microarray data were processed and analysed using the language R/BioConductor (R Development Core Team 2008 [46]). Quantile normalization was performed on background-corrected features with the limma package [47]. Arrays having more than 3 % of not uniform features were eliminated for subsequent analysis. Filtering step was performed according to Agilent Feature Extraction software results on spot quality and spot intensity reliability. Negative filtered features were excluded from subsequent analyses. Missing values were imputed with impute package [48]. Raw and normalized hybridization values are deposited in the gene expression omnibus (GEO) repository with the accession number GSE66103.

We first applied a redundancy analysis (RDA), a constrained ordination method implemented in the Vegan package [49], to obtain a global view of the extent to which the explanatory variables “sex” (male, female) and “progeny” (Denmark, France, hybrid) influenced the expression levels. Three RDA were performed. The first one was a full model with sex and progeny as explanatory variables (Sex + Progeny). Then, two partial models were applied in order to partitioning explicable variance on sex and progeny (respectively: Sex + Condition(Progeny) and Progeny + Condition(Sex)). Contributions of genes to RDA axis 2 were retrieved in order to identify individual transcripts that contribute the most. Secondly, differentially expressed transcripts between French and Danish progenies were also identified with analysis of variance. Fixed factors for the two-way ANOVA were sex and progeny. Multiple testing *p*-values were adjusted using Benjamini-Hochberg (BH) correction. Finally, a scan of phenotypic differentiation of expression levels was performed (eP_ST_ scan). Additivity on all mRNA expression levels was specifically evaluated by assessing if hybrid expression was not different from the theoretical mid-parent level (tested by t-test at *p*-value < 0.01) as proposed by Hedgecock et al. [50]. Then eP_ST_ estimates were calculated using the equation: σ^2^_GB_/(σ^2^_GB_ + 2σ^2^_GW_), where σ^2^_GB_ and 2σ^2^_GW_ represent among- and within-population components of the genetic variance for quantitative traits respectively [33], and tested by permutations (*N* = 5000). Transcripts for which the eP_ST_ values exceeded the 0.999 quantile of the permutation distribution were retained as outliers. The script used for RDA, ANOVA and eP_ST_ calculations is in Additional file 1.

Transcripts putative annotations were identified using ngKlast blast (Korilog) against a protein data base (*E*-value 1.0 × 10e^−5^) obtained from the *C. gigas* sequenced genome and transcriptome deposited on Genbank [51]. GO terms were obtained using ngKlast against the Swissprot database (E-value 1.0 × 10e^−5^). GO terms enrichment analysis were performed using the Fisher’s Exact test on Blast2Go [52]. Hierarchical clustering was performed using Ward method and 1-correlation as dissimilarity matrix.

### Statistical analyses of sex-ratio and condition index

Data for sex-ratio, condition index and biochemical analyses were processed and analysed using the language R/BioConductor [46]. Comparisons of sex distributions between progenies were made using Chi-square tests (*Χ*^2^). The condition index, was tested using a two-way ANOVA with sex and progeny as fixed factors, post-hoc test (LSD test) was used to determine which groups were significantly different. Normality was checked using Shapiro-Wilk test and homogeneity of variances matrices with Bartlett test.

## Results

### Sex-ratio and condition index

**Fig. 1:**
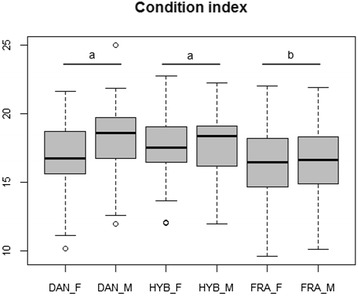
Boxplot of the condition index. Condition index by progeny and sex, small letters indicate progeny effects found by the LSM post-hoc test. DAN = Danish, FRA = French, HYB = Hybrids, F = Females, M = Males (*N* = 27-31)

Sex-ratio differed significantly (*p* <0.01) between French and Danish progenies with a *female*-biased sex-ratio observed in the Danish progeny (40 %) compared to the French progeny (73 %). Hybrids presented an intermediate but not significantly different sex-ratio (58 %) from French and Danish progenies. The two-way ANOVA for the condition index (CI) showed a progeny effect (*p* <0.003). The condition index of the 3 progenies clustered into two significantly different groups, hybrid and Danish progenies showing significant higher values (17.5 %) than French one (16.4 %, Fig. 1). Histological examination showed that all individuals of the three progenies were in stage 3, corresponding to full ripeness.

### Gene expression analysis

**Fig. 2:**
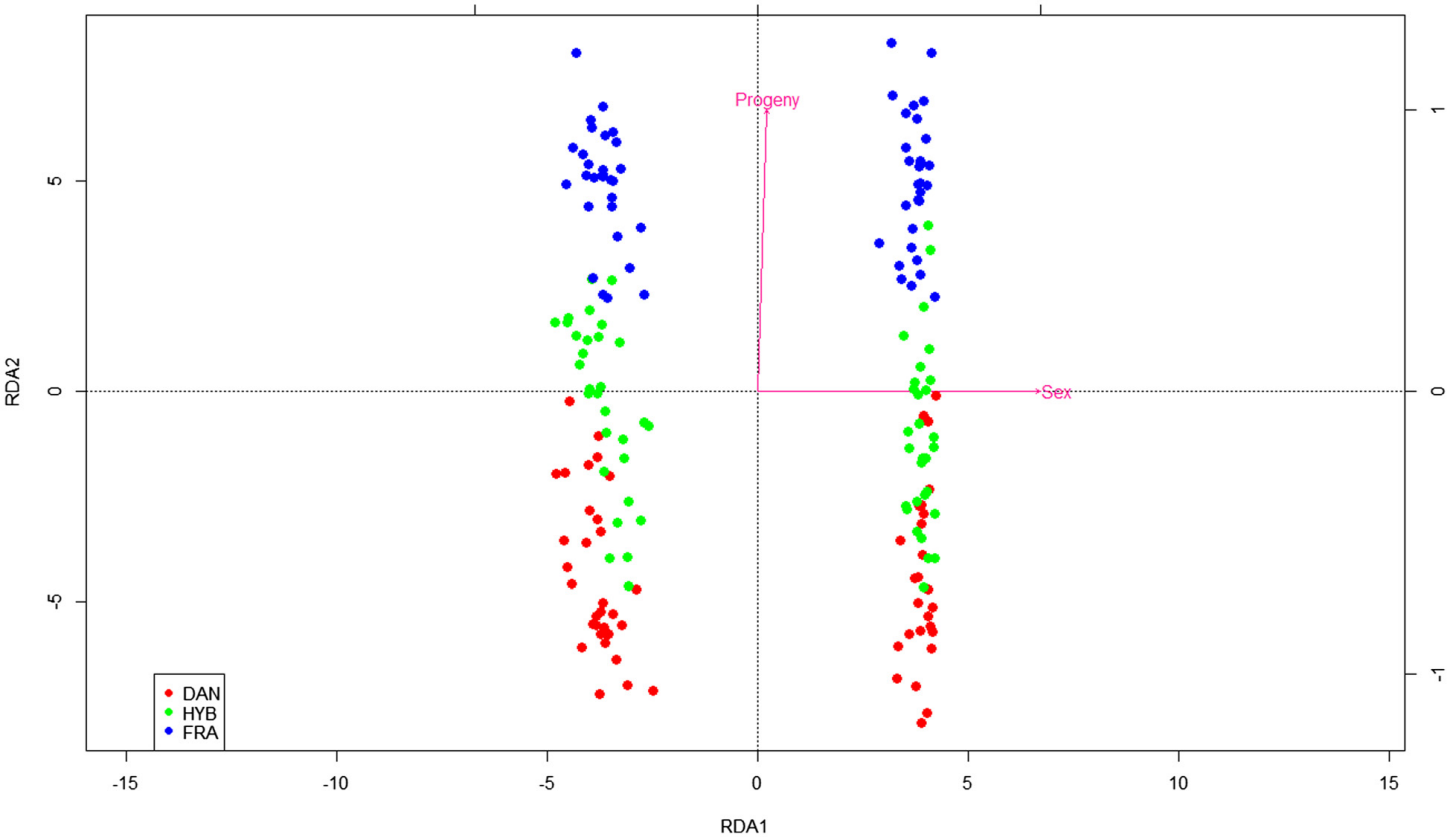
Plot of RDA full model. Each point corresponds to an oyster sample characterized by its gonad microarray gene expression, sex represent 95.5 % and progeny 4.3 % of the explained variance. Female are represented on the left side of the plot and males on the right. DAN = Danish, FRA = French, HYB = Hybrids

**Fig. 3:**
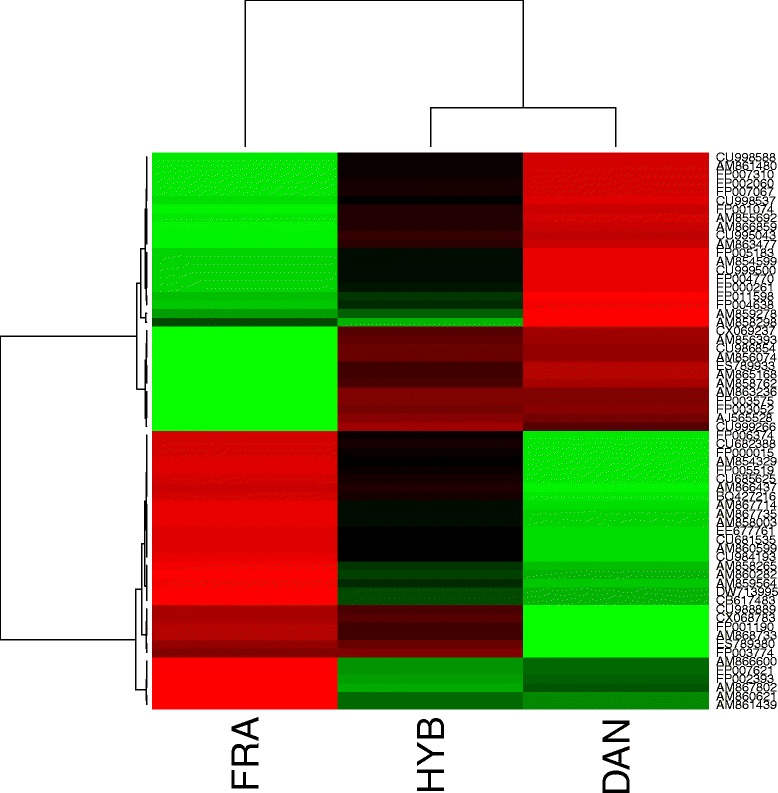
Heatmap of RDA progeny significant transcripts. 64 transcripts whose contribution accounted for the 0.1 and 99.9 percentile of the RDA2 axis distribution scores; each column represent the averaged mRNA expression for French (FRA), hybrid (HYB) and French Danish (DAN) progeny (*n* = 58–60). Clusters were obtained using Ward method and 1-correlation as dissimilarity matrix

RDA analysis on all mRNA and individuals clearly discriminated males from females on its first component RDA1 (Fig. 2). From the two following partial RDA, sex accounted for 95.5 % and progeny for 4.3 % of the explained variance, respectively. The two explanatory variable vectors were orthogonal on the RDA plan, and hybrids were intermediate between French and Danish progenies on second component RDA2. Scores of contribution to second component RDA2 allowed us to obtain a first list of 64 genes whose contribution accounted in the 0.1 and 99.9 percentile of the distribution. Genbank access, putative annotations and E values of these transcripts are presented in Table 1 and their mean expression *per* progeny in the heatmap in Fig. 3. The enriched GO biological processes attributed to those genes are presented in Additional file 2.

**Table 1:** RDA sex significant transcripts 64 transcripts whose contribution accounted for the 0.1 and 99.9 percentile of the RDA2 axis distribution scores; Genbank accession number, putative annotations, E values, cluster and presence on these transcripts in the other analyses (a = ANOVA, m = eP_ST_ males, f = eP_ST_ females)

Genbank	Hit definition	E value	Cluster
CU998588			1
AM861480	hypothetical protein CGI_10017906	1.00E–17	1
FP007310			1
FP002060			1
FP007067			1
CU998537			1
FP001074			1
AM855692	hypothetical protein CGI_10011429	6.00E–53	1
AM866859	A–kinase anchor protein 7 isoform gamma	2.00E–70	1
CU995043	Cadherin–23	2.00E–25	1
AM863477	Barrier–to–autointegration factor	2.00E–08	1
FP005183			1
AM854599			1
CU999500			1
FP004770	Zinc finger protein 26	3.00E–12	1
FP000261	CD63 antigen	3.00E–106	1
FP011598			1
FP004638			1
AM859278			1
AM858298	RING–box protein 1	6.00E–42	1
CX069237	Actin	8.00E–38	1
AM856393	hypothetical protein CGI_10011429	1.00E–31	1
CU986854			1
AM856074	hypothetical protein CGI_10023286	4.00E–113	1
ES789933	Coatomer subunit gamma–2	3.00E–10	1
AM865168			1
AM858762	hypothetical protein CGI_10024669	6.00E–35	1
AM863236	Transaldolase	3.00E–86	1
FP003575			1
FP003052			1
AJ565528			1
CU999266	hypothetical protein CGI_10005871	1.00E–42	1
FP006374	Interferon–induced, double–stranded RNA–activated protein kinase	5.00E–10	2
CU682388	Ephrin type–A receptor 2	3.00E–25	2
FP000015	Glutathione reductase, mitochondrial	2.00E–12	2
AM854329			2
FP005519			2
CU685625	Galectin–4	2.00E–43	2
AM866437			2
BQ427216	tRNA 2′–phosphotransferase 1	1.00E–06	2
AM867714			2
AM867735	Ribosomal RNA–processing protein 8	1.00E–39	2
AM858003	Mortality factor 4–like protein 1	6.00E–101	2
EE677761	L–rhamnose–binding lectin CSL3, partial	6.00E–46	2
CU681535			2
AM860599	hypothetical protein CGI_10018932	3.00E–40	2
CU984193	hypothetical protein CGI_10008458	7.00E–15	2
AM858265			2
AM860282			2
AM859564			2
DW713995	Dedicator of cytokinesis protein 6	2.00E–32	2
CB617483	hypothetical protein CGI_10016110	8.00E–11	2
CU988889	DNA polymerase epsilon subunit 4	1.00E–58	2
CX068783	hypothetical protein CGI_10013277	4.00E–26	2
FP001190			2
AM868733	Anaphase–promoting complex subunit 13	4.00E–39	2
ES789380	hypothetical protein CGI_10020002	8.00E–72	2
FP003774	Multimerin–1	3.00E–10	2
AM866600	DNA topoisomerase 1	3.00E–54	2
FP007621			2
FP002393			2
AM867802			2
AM860621			2
AM861439			2

The two-way ANOVA performed on French and Danish progenies confirmed the strong sex effect on gene expression already identify with the RDA, with 11,472 differentially expressed transcripts between sexes, and a progeny effect with 80 transcripts differentially expressed (BH correction, cut-off *p*-value of 10^−4^ presented in Additional file 3). Twenty of these 80 genes were in common with the candidates identified with the contribution to RDA2.

Due to the strong differentiation in gene expression between males and females, the eP_ST_ were estimated separately by sexes on all transcripts. Only 953 and 980 transcripts respectively (corresponding to 6 % of the total 31,918 *C. gigas* transcripts present on the microarray), showed an mRNA level intermediate in hybrids between the French and Danish progenies. Average eP_ST_ values were 0.04 for both males and females. Transcripts considered as outliers had eP_ST_ values higher than 0.15 for females and 0.17 for males. The number of outlier transcripts was 53 on males and 69 on females, and they are presented in Additional file 4. Ten transcripts were in common between all analyses, two of these having known putative annotations (A-kinase anchor protein 7 isoform gamma and Galectin-4).

## Discussion

So far, colonization of *C. gigas* on European coasts has been investigated by ecological or marker-based population genetic approaches, but the variability of phenotypic traits potentially associated with this range expansion has only very recently been investigated [7]. Despite strong difference in expression levels between sexes, as previously reported [53], we nonetheless detected a clear difference in expression levels between progenies of oysters originated from France and Denmark, and their hybrids. From the RDA approach on microarrays we found that 4.3 % of the explained variance is ascribed by the progeny in a mainly orthogonal way to sex differences, which means that despite the huge transcriptomic differences between sexes, the more modest differences between progenies proved to be mainly independent of the sex. Only characters being heritable could allow adaptation because significant genetic bases of trait variation (rather than only phenotypic plasticity) are necessary to allow response to eventual natural selection. In our study, the use of hybrid progeny allowed to identify transcripts presumed to show additive genetic variation. In the RDA the hybrid progeny appeared intermediate between French and Danish, and 50 % of the transcripts contributing the most to RDA2 axis proved to behave mainly additively. Our analyses allowed us to obtain a list of candidate genes, the expression of which might have reflected adaptation during invasion or were already genetically differentiated in the founding populations. By the way overall microarray features, only a very limited proportion of the total genes showed intermediate values in hybrids. The extensive non additivity of the transcriptome has been observed in Drosophila (Gibson et al. <2 %, [54]) and maize (Auger et al., ~30 % [55]) in contrast with the classical assumption in quantitative genetics of predominately additive genetics effects. Furthermore, a low proportion (2 %) of additive patterns of gene expression was previously observed by transcriptomic analysis in larvae of partially inbred Pacific oyster populations [50]. Finally, another study on oyster showed the non-additive nature of genetic variance for fitness-related traits and that the non-additive genetic component of yield is often the largest [56]. However, by focusing on a population likely to have adapted to a new environment during invasion (Denmark), we have access to the adaptation filter on phenotypic evolution and uncover additively behaving traits (intermediate expression in hybrids) in the subset of transcripts that are the most differentially expressed. Interestingly, Wendling and Wegner [7] recently investigated the adaptive potential of North Sea *C. gigas* populations to local Vibrio spp., proposing that dominantly inherited resistance could facilitate fast adaptation.

In our study, ANOVA and eP_ST_ analyses gave complementary results on phenotypic traits differentiation between the Danish and the French progenies. The majority of the mRNA differentially expressed in the ANOVA analysis was not in common with eP_ST_ analysis. This could be explained by the fact that eP_ST_ estimates were only performed on transcripts showing additive patterns and that most of the eP_ST_ outliers did not have a high p-value in the ANOVA analysis. Roberge et al. [37], who find similar discordant results between ANOVA and Q_ST_ estimates on salmon, suggested evolution as consequence of selection but without an additive genetic basis for these traits. eP_ST_ estimates performed separately on males and females, demonstrates that considering sexes separately, when sex effect is so strong on gene expression patterns like in in gonads, could help in highlighting new candidates. Furthermore, this suggests that sex-dependent adaptation might be involved in the observed genetically-based phenotypic and transcriptomic variations of reproductive traits, supported by an increasing evidence of a role for sex differentiated effects in the architecture of complex traits [57]. Sex interaction effects are common in model organisms for a wide range of traits, and can often explain a substantial part of the genetic basis of phenotypic variation [58].

In males, functional annotation of outlier eP_ST_ estimates pinpointed mRNA encoding proteins involved in sperm motility. The A-kinase anchor protein 7 isoform gamma [Genbank:AM866859], identified as outlier eP_ST_ both in males and females and more expressed in Danish progeny, is a A-kinase anchoring protein (AKAPs) promoting the selective sequestration of intracellular cAMP-dependent protein kinase (PKA) involved in epithelial sodium channel regulation [59–61].This protein has been suggested to have additional and perhaps unique function in spermatozoa as a scaffolding protein for the Rho-GTPase pathway that regulates sperm motility. Furthermore, Kington et al. [62] found high expression levels of proteins having an important role in sperm motility of *C. gigas* involved in Rho signalling pathways. Sperm motility is commonly used as criteria of male gamete quality linked with the fertilization success and therefore potentially favouring colonization of new habitats. Furthermore, Filamin-A [Genbank:CU686267], more expressed in Danish progeny, is an Actin-binding protein that participates in the anchoring of membrane proteins for the actin cytoskeleton and is involved in sperm morphogenesis in mammals [63]. Finally, as cells protection against oxidative injury in marine invertebrates [64], Alternative oxidase (AOX, [Genbank:BQ426710]), more expressed in Danish progeny, may have a role in oxidative protection in gonads, and potentially in gamete quality. In females, the Molluscan insulin-related peptide 5 [Genbank:CU987248] was more expressed in Danish progeny. In mammals, the role of insulin pathway in fertility is well known [65] and in fish the insulin pathway has been positively associated to gamete quality [66]. In *C. gigas*, an Insulin-related peptide receptor has previously been identified in oysters by Gricourt et al. [67] as well as several factors of the insulin signalling pathway. Jouaux et al. [68] found that insulin pathway elements can modulate germinal cells proliferation during food deprivation in the first stages of gametogenesis with expected consequences on fertility.

A comparison of divergence in neutral markers, F_ST_, to divergence in phenotypic traits, Q_ST_, has widely been used as a method to assess the relative strength of genetic drift and selection [34]. The Q_ST_ levels typically exceed that observed in F_ST_ suggesting an important role of natural selection on quantitative traits [34, 69]. In our study, we did not have access to Q_ST_ values but we restricted P_ST_ values to additively behaving transcript with intermediate expression levels in hybrid. We found that mean eP_ST_ (0.04) was similar to the estimated F_ST_ values [24] on the same populations, while the outliers eP_ST_ had values greater than 0.15. Our results therefore suggest that diversifying selection has most probably acted on outlier gene expressions. Finally, gene expression can also be controlled by epigenetic mechanisms, potentially in a transgenerational manner, meaning in a way, by genetic mechanisms non-DNA dependents. There are yet only a few papers studying epigenetics in oysters [70, 71] and this is clearly an emerging topic. Moreover, few studies until now focused on epigenetic-mediated adaptation in invasive species [72].

### Sex-ratio and population expansion

In our study, we observed a female biased sex-ratio in the Danish progeny. Sex-ratio biased to female in invasive species has also been observed in an estuarine shrimp, *Palaemon macrodactylus* [73] and was proposed as a good descriptor to detect invading populations in signal crayfish, *Pacifastacus leniusculus* [74]. Although sex determinism in *C. gigas*, an alternative and irregular protandrous hermaphrodite, is complex, it seems to be determined both by environmental and genetic factors [75]. In our study, environmental effects were minimized by rearing progenies in common garden conditions, indicating that the observed differences in sex-ratio are genetically based. However interactions with environmental conditions have been suggested to result from regulatory pathways involved in sex determination [76]. Two genetic models have been proposed for sex determinism in *C. gigas*. The first suggests the presence of 2-genotypes, a dominant male M allele and a protandric recessive F allele [77]. The second propose a 3-genotypes model, FF for true female oysters, MM for true male oysters and FM for individuals that may mature as females or males [75]. Furthermore, an energy-mediated sex determinism was proposed [78], in which sex-ratio could be a possible way to select faster-growing populations when more females are produced in the first year of the life. The greater condition index observed in the Danish progeny could translate a greater reproductive effort in this new-expanded Danish population. This index is strictly correlated to ripeness and gonadal occupation production during gametogenesis in oysters [79]. In species like *C. gigas*, having an “r” demographical strategy, characterized by high fecundity, reproductive success is greatly dependent on the quantity of gametes produced, especially oocytes, as well as their quality. Furthermore, Cardoso et al. [80] found that in Northern European locations, oysters produce smaller eggs in larger quantities, suggesting an increasing reproductive output. The authors proposed that, since smaller oocytes are thought to have a longer development time, the environmental conditions along the Northern European coasts may result in increased larval dispersal and possibly in further population expansion. In this context, a greater reproductive effort together with a female-biased sex-ratio in *C. gigas* could favour a rapid colonization of new habitats.

## Conclusions

To conclude, genetic differentiation previously reported between *C. gigas* Southern Europe populations and population north to the Wadden Sea is corroborated by phenotypic differentiation based on transcriptomic data, biased sex-ratio and condition index. Overall, our results suggest a population expansion strategy of the studied Pacific oyster Danish population, potentially relying on a females-biased sex-ratio, a greater reproduction effort and gamete quality, noticeable on molecular signatures.

### Data availability

Microarray data are deposited in the gene expression omnibus (GEO) repository with the accession number GSE66103 (http://www.ncbi.nlm.nih.gov/geo/query/acc.cgi?acc=GSE66103).

### Ethics statement

All the experiments were conducted according to the regulations of local and central government, and the study protocol was conducted in accordance with institutional guidelines

## Additional files

Additional file 1:
**Script used for RDA, ANOVA and eP**_**ST**_ **calculations.** (R 10 kb) Additional file 2:
**GO terms of RDA sex significant transcripts.** GO associated terms for transcripts whose contribution accounted for the 0.1 and 99.9 percentile of the RDA2 axis distribution scores. (XLS 179 kb) Additional file 3:
**ANOVA sex significant transcripts.** 80 mRNA differentially expressed in the two-way ANOVA between French and Danish progeny, Genbank accession number, putative annotations E values and GO associated terms. (XLS 132 kb) Additional file 4:
**eP**_**ST**_ **outliers for male and females oysters.** 53 and 69 outliers eP_ST_ mRNAs for male and female oysters respectively, Genbank accession number, putative annotations, E-values, fold changes and GO associated terms. (XLS 160 KB)
